# Strychnia as a Poison

**Published:** 1874-08

**Authors:** J. Q. A. Hudson


### STRYCHNIA AS A POISON.

BY J. Q. A. HUDSON, M.D.

*Read before the Meigs & Mason Academy of Medicine, Feb. 26, 1874.*

"We know of no cases in which, from the history of the symptoms alone, a more certain inference can be drawn as to their origin, than it can be in such as exhibit the phenomena of strychnine-poisoning in its most characteristic form."—*William B. Carpenter, author of several works on Physiology. B. & F. M. C. Review. October*, 1856, *p.* 340.

The use of strychnine as a poison, either for the purposes of suicide or murder, or from accident, has become so frequent, that it becomes important that physicians and jurists should be thoroughly informed as to the distinctive characteristics of strychnine-poisoning, not only in its ordinary features, but also in all its variable phenomena, as influenced by variations in dose, by combination with other drugs, and by co-existing conditions of the stomach, or of the general system.

Strychnine-poisoning has been often mistaken for disease, much oftener than disease has been been mistaken for strychnine-poisoning. Before the characteristic phenomena of strychnine-poisoning became thoroughly investigated and generally known, it is quite certain that many instances of strychnism in the human subject, were mistaken for tetanus, and were treated for that disease. On this point Alfred S. Taylor says:

"Up to 1856, there had been a large number of fatal cases of tetanus; the facts had been accurately recorded, the mortality noted, and no one had thought of analyzing the contents of the stomach in these cases, for the simple reason that the labor would have been superfluous. On the other hand, it is by no means improbable that some poisonings by strychnine, may have been passed over as idiopathic tetanus; and of this the case of Miss Abernethy furnishes a striking example."—*On Poison, p.* 137.

Since the introduction and universally extensive employment of strychnine as an invaluable remedy, its effects have been the subject of careful experiment and observation by men of acknowledged ability, and its action, physiological and toxic, upon man and animals is now well known, so that any one who will take the time and trouble to obtain information on this point, need not remain ignorant.

A great incentive to the acquisition of this knowledge, arises from the fact that in the defense of those who are charged with the crime of administering strychnine with poisonous intent, and where they are really guilty of the crime, legal counsel will use every means to cover up the truth, and ignore the facts of science, in order to screen his client from guilt and avert the just penalties of the law.

Attempts will be made to induce the court and jury to believe that the symptoms exhibited, though plainly indicating to the medical expert the action of strychnine, are the result of tetanus, or of some lesion or disease of the brain, or spinal cord, or of the general nervous system.

The truth must also be told, though its exposure is a professional disgrace that medical men may be hunted up, who are ready, in the words of Prof. A. S. Taylor, to "trade in evidence," and are willing to lie under oath, and trample on science, for the sake of pecuniary remuneration, clandestinely, though not the less actually received. The honest physician should be prepared as a medical expert, to meet properly and successfully the false testimony of such quasi scientific villains.

It may be presumed, however, that while we have a class of the legal trade who hold the unenviable, and too often very questionable moral position, styled criminal lawyers, we may expect that in criminal cases requiring medical testimony, there will be found medical men, of third and fourth rate medical learning, and possessing no solid, appreciable rate of moral honesty, ready for paltry gold to make a false oath.

This false testimony is carefully treasured in the law reports, and is again and again brought out by the criminal lawyer to help him in like cases.

What a beautiful record do these perjured medical liars make for themselves! They justly merit, and will surely receive, the scorn and contempt of the present and coming generations of scientific men.

As the degraded criminal lawyer, who in the defense of his guilty client, when employing fabricated debris and other manufactured testimony, is despised by the honest, respectable lawyer, so the perjured medical witness, who aids him to construct his refuge from crime, will forever be spurned and despised by the good and true of the medical profession.

### *SYMPTOMS OF POISONING BY STRYCHNIA.*

In most cases, within an hour after strychnia is taken by the stomach, the symptoms of the action of the drug are developed. If the dose is moderate, or if the form of the poison is of a nature not readily soluble, as the powdered nux vomica, the first indications are those usually of general uneasiness with a feeling of suffocation, which sooner or later are followed by the peculiar systemic action of the poison.

If the dose has been large, or if large and in a soluble state, or in a state of solution, the symptoms of the strychnine are from the inception nearly always rapidly evolved. These consist of tremblings of the whole body, accompanied, or occuring alone, by jerkings and twitchings of the muscles, which, by most observers, have been compared to electric shocks. These electric twitchings are mostly confined to the muscles of the trunk and lower extremities, particularly the latter, the arms being rarely affected.

The patient is in a state of mental anxiety, and forebodings of a fatal result are expressed.

A general tonic spasm of a tetanic character soon supervenes, coming on suddenly, and involving the muscles of the whole body; but more marked in the trunk and lower extremities.

The spasm is generally opisthotonic, the head, neck, trunk and lower limbs being curved backwards, and if the position of the body is supine, it rest upon the occiput and heels. During the spasm the body is fixed and rigid, the muscles firmly contracted and as hard as a board. The contraction of the muscles are powerful, varying in intensity in different cases, oftentimes occasioning rupture of the muscular fibre.

The muscles of respiration being involved, this function (respiration) is almost wholly suspended, and probably in some cases is entirely so.

The heart beats rapidly and with irregular, tumultous action, the pulse being small and rapid, so much so that sometimes it is impossible to number the pulsations. The partial suspension of respiration, induces a stasis in the vessels of the cranium and face, giving the latter a dusky, livid appearance, great or less, according to the degree congestion produced. These are symptoms of asphyxia.

The eyes are staring, fixed and prominent, though in some few cases they are turned upward.

The jaws during the paroxysm are usually set as in tetanus, though in a few cases this does not occur, even during the paroxysm. (Taylor.)

The sardonic grin, *risus sardonicus,* is sometimes observed, though this is often absent.

The spasmodic contraction of the muscles causes excessive pain.

Profuse perspiration generally occurs during the later paroxysms, when symptoms of debility arise.

The duration of the paroxysm varies in different cases, from a half a minute to eight minutes, the latter being the extreme. The duration is usually less than two minutes.

After the spasm subsides, the patient lies in a state of exhaustion.

Profuse perspiration occurs proportionate to the debility. Respiration becomes easier, deglutition can be performed, and the trismus, if existing during the spasm, is relieved. After a longer, or shorter time, the peculiar electric twitchings reappear, to be followed by other paroxysms.

Taylor states without qualification, that in fatal cases the paroxysms increase in duration and severity till death, while Stille says that they may or may not be severe in the later progress of the strychnism.

The paroxysm, in some instances, comes on with such suddenness and violence as to throw the patient off the bed.

It is stated that the pupils are dilated during the paroxysm, but are contracted during the interval. Whether this is invariably true is not known. I think it probable, that in cases accompanied with excessive asphyxia, the pupils are dilated during the spasm, but I question if there is much more than the natural contraction of the pupil during the interval. Further observations are required to decide this point.

The patient is perfectly conscious during the interval, and the mind seems to be unaffected, except by the fearful forebodings of impending dissolution, and the fear of the approach of another paroxysm of which he is cognizant, and will often ask to be held when it is coming on. It is stated by several observers, that the paroxysm of strychnine tetanus, is universally ushered in by screams and cries of the patient; and I have given this on the authority of several writers, as a diagnostic feature of strychnine-poisoning.

The muscles of the trunk and neck are more or less rigid and contracted during the interval as in tetanus, and many of the phenomena of strychnism are similar in every respect to those of tetanus. The progress of the case is always more rapid, however, and Taylor states, that in strychnism the case, within two hours from the commencement of the paroxysms, usually terminates in death, or the violence of the symptoms abating, recovery ensues. Death arises either from asthenia or exhaustion during the interval, or during a paroxysm from asphyxia from the powerful contraction of the muscles of respiration, or of the glottis, impeding respiration.

There is in strychnism, as in tetanus, hyperæsthesia of all the sensory nerves, extending often to the organs of special sense, so that the slightest touch, a breath of air, or a sudden noise will excite a paroxysm.

This hyperæsthesia of the excito-moto system is probably more prominent than in tetanus. In some cases of tetanus it is absent, or exists in a slight degree, while it is believed to be *always* present in strychnism.

These are two points in the history of cases of strychnine-poisoning, that are of great importance, in a medico-legal point of view. These are:

1. The time that may elapse *before* the occurrence of tetanic paroxysms; and,

2. The time that may elapse before death *after* their development.

The history of recorded cases will aid in giving a correct answer to these questions. There are certain conditions that influence the *time* of the action of this as well as of other poisons, and other conditions that modify the *duration* of action. These are:

1. The *form* of the poison, whether in state of solution, or in a soluble or insoluble form. This influences the promptness of action.

2. *Repletion* or *emptiness* of the stomach. This modifies the time likewise.

3. A *torpid* or *active* state of the stomach, from whatever cause. This modifies the time.

4. *Individual susceptibility.* This modifies time of action, and probably the duration of action. This individual susceptibility is illustrated in the daily professional use of strychnia as a remedy, requiring at times doses to produce a physiological or remedial effect, that would be toxic in another person.

5. *Mode* of *administration* and the amount of dose administered. These will modify the time of occurrence, as well as the duration of the symptoms of strychnism.

Hammond very justly remarks on this point as follows:

"It is well known that strychnia is not unfrequently employed for the purpose of committing murder or suicide, and it is possible so to employ it for either of these purposes, as to cause its effects to extend over a long period of time, and thus to add to the difficulties attending the discrimination. Even in such a case, however, the diagnosis can be made, if due care and a thorough inquiry into the case be made." Again he says:

"In those cases of poisoning by strychnia, in which the doses are small, and administered at comparatively long intervals, the symptoms are mitigated in violence, and consequently one of the distinguishing features of the two affections is lost. Still, the general character and sequence of the phenomena are the same, and it is not probable that careful observation and inquiry will fail to elicit the true nature of the case."—*Diseases of the Nervous System, p. p.* 535 and 586.

6. The action of strychnine is very materially modified by other drugs, given in combination, or after it has been administered.

It is not improbable that some persons who use strychnine with suicidal or murderous intent, will use means to inform themselves of the peculiarity of action, and will attempt in every possible way to modify the characteristic symptoms of the poison, so that the affection may not be recognized. This may be done as stated by Hammond, by using small doses at long intervals, or by using other drugs, either at the same time or after the strychnine is developed.

In the notorious case of Cook, poisoned by Palmer, antimony was employed before the strychnine was administered.

Opium or its salts, given with strychnia, delay the time of development of the poisonous action of the latter, as well as prolong life, and, probably, in some instances, if the quantity of opium is sufficiently large, overcome the action of strychnia to such an extent as to insure recovery. Stille says:

"In a case reported by Mr. Chippendale, four grains of morphia and as many of strychnia were taken, and, very probably, the morphia may have acted, rather than the charcoal subsequently administered to prevent, or at least delay, the poisonous effects. Such, in fact, was what occurred in direct experiments made by Rifoldo Rifaldi. A grain of strychnia was given to a dog, and in a few minutes a grain of morphia, also; the symptoms were slowly developed, and slight, and the dog recovered. A similar result was long since obtained by Pellitier and Caventon."—*Materia Medica and Therapeutics, Vol.* 2, *p.* 251.

It may be stated that the time of the accession of paroxysms of tetanus, after taking the poison, is about twenty minutes, though there are many instances in which they took place in five minutes, and many in which they did not appear for one, two or more hours. Without going into detail, by referring to or quoting cases, it may be stated that many instances are recorded in which tetanic convulsions were not developed for one, two, three or more hours. In one instance five hours elapsed. A man procured two powders, 3½ grains each, of strychnine, the druggist supposing he was giving him morphine. The man was accustomed to taking large doses of morphine for facial neuralgia. He took one dose (3½ grains of strychnine) at the druggist's. No symptoms occurred except a feeling of coldness and numbness in the lower extremities and lower part of the back. Five hours afterwards, as he was about retiring for the night, and being apprehensive of the tic, he took the other powder, (3½ grains.) A few minutes after which general strychnine tetanus came on. Under appropriate treatment he recovered; and it is stated his neuralgic symptoms entirely disappeared.—*American Journal of Medical Science, April,* 1848, *p.* 562.

The length of time elapsing till death from the appearance of general spasms, is usually within two hours; but the period may be made shorter or longer. Death is speedily produced by the extreme violence of the paroxysms in some cases. Taylor makes this statement: "In a case privately reported to me, ten grains of strychnia, given by mistake for sulphate of quinine, killed a patient in ten minutes."—*On Poisons, p.* 683.

Dr. Warner took by mistake half a grain of strychnia. The symptoms commenced in five minutes, and he was dead in twenty minutes.—*Taylor on Poisons, p.* 687.

Taylor also gives instances of prolonged periods before death. A child lived three and a half hours after development of spasms. The dose was one-sixteenth of a grain.

A man took three grains of strychnia. In three-quarters of an hour tetantic spasms occurred, and five and one-fourth hours from the occurrence of the paroxysms he died.

It is undoubtedly a fact, that in many cases of poisoning by strychnia, when given for purposes of murder, that if it is found that a certain dose administered, although producing toxic action, does not cause death, a second, third or even more doses are given of the same or greater quantity as the first. Thus, in the history of the these cases, we have a succession of periods of strychnism, extending over a longer or shorter extent of time.

Thus in the case of John Freeman, poisoned by his concubine, a Miss Smith, he had several attacks of strychnine tetanus, following the administration of pills. One on Friday, another on the next day at 10 P. M., and a third fatal attack on the following Monday.

We have a similar history in the repetition of strychnia convulsions at intervals, and following soon after the administration of the drug, in the cases of Cook, poisoned by Palmer, and of Mrs. Dove, poisoned by her husband.—*See Taylor on Poisons, p.* 695, *et sig., and Brit. and For. Med. Clin. Rev., p.* 409, *case IX.*

*Fatal dose.* The variations in individual susceptibility, and the variable conditions influencing the action of the poison, which have previously been stated, very greatly affect the dose required to produce fatal results.

Small doses have produced dangerous symptoms and even death; and very large doses, as a medicine, have been used without dangerous effects. Thus: Six-twentieths of a grain, in doses of one-twentieth of a grain, and extending over a period of two days, caused tetanic spasms.—*Taylor's Med. Jur., p.* 171.

I have mentioned the case of Dr. Warner, in whom half a grain caused death, and another case in which recovery followed the taking of seven grains.

Taylor gives the fatal dose of strychnia at from a half to two grains. No definite fatal dose is given by Taylor, for ext. nux vom., powdered nux vom., or any other drug containing the poison.

*Diagnosis.* The only affection, that in the least approaches in resemblance strychnia-poisoning, is tetanus. The similarity is very complete; yet, by noting carefully the peculiar symptoms belonging to each affection, and especially by observing the history of the symptoms from the commencement, the physician will rarely fail to make a correct diagnosis. The following are the principal points of differentation:

1. A premonitory symptom, which I believe is invariably present in tetanus, is an intermittent spasmodic pain, shooting from the precordia or ensiform cartilage to the back. There is constant precordial distress with the spasmodic pain, which comes and goes at irregular but rather brief intervals. This symptom continues during the entire period of the disease. This spasmodic pain, *as a prodrome* of strychnine-poisoning, is never seen. There may be severe pain, caused by spasmodic contraction of the diaphragm, during the paroxysms of strychnine tetanus, but as a forerunner of the paroxysm, it is believed never to occur. Hammond gives this as an important diagnostic of tetanus.

1. Tetanus is always preceded by a stiffness of the muscles of the neck and lower jaw. The lower jaw cannot be fully opened, and sometimes before the tetanic convulsions supervenes, the jaw becomes firmly set and cannot be opened by any reasonable force. This stiffness of the neck and jaw, as well as the precordial pains, sometimes precedes for hours the occurrence of the general spasm in tenanus. On the other hand, in strychnine tetanus, *trismus* does not exist, except *during the paroxysms*, and even then it is sometimes absent. Taylor says, in describing the symptoms of poisoning by strychnine: "The jaw is not primarily attacked, and is not always fixed during the paroxysm."—*Med. Juris., p.* 169.

3. *In tetanus*, deglutition is performed with difficulty, or may be wholly impeded or impossible during the *suspension of the paroxysm.* In strychnine tetanus, during the intervals, there is not very great difficulty in swallowing, though it is not done with the same ease as in health, and is performed in a sudden, gulping manner.

4. The approach and accession of a spasm of strychnine tetanus is accompanied by sudden cries and screaming. In true tetanus, these expressions of distress are absent.

5. In strychnine tetanus, the entire muscular system is involved during the paroxysm, including the upper and lower limbs, and even the hands and feet. In tetanus, the contraction of the muscles of the limbs is not so severe, and is sometimes absent; the hands are rarely affected, and in many instances the arms are unaffected.

6. The electric shocks, or muscular twitchings, are peculiarly diagnostic of strychnia-poisoning when present. They may not arise until after the first general spasm, and if the patient survives it, they come on before the supervention of the second. These electric shocks are not observed in tetanus.

7. The progress of the symptoms in strychnine-poisoning is very rapid, the paroxysms succeeding each other with great rapidity, and, save in exceptional cases before referred to, the affection terminates in death or amelioration within two hours. In tetanus, the course is comparatively more gradual, there elapsing several hours or a day or more, from the inception of the initiatory symptoms of epigastric pain and stiffness of the muscles of the neck and lower jaw, before occurrence of tetanic convulsions. The paroxysms of tetanus occur at longer intervals, and death, in the most rapidly fatal cases, does not take place for several hours. There are a few remarkable exceptions to the last statement, as death has been known, in one instance at least, to have occurred within fifteen minutes after an injury.—*See Hammond's Dis. of Nervous System, p.* 532.

I have now given the diagnostic characteristics of strychnine-poisoning. The symptoms, which are similar in tetanus, I need not dwell upon. They are the muscular rigidity during the intervals, except that of the lower jaw, the general sensitiveness of the excito-motory nerves, the symptoms of asphyxia during the paroxysm, and the state of the pulse.

The physician who can obtain a fairly correct history of a case of either affection, will rarely fail to make a correct diagnosis.

